# Processing of LtaS restricts LTA assembly and YSIRK preprotein trafficking into *Staphylococcus aureus* cross-walls

**DOI:** 10.1128/mbio.02852-23

**Published:** 2024-01-04

**Authors:** Amany M. Ibrahim, Muhammad S. Azam, Olaf Schneewind, Dominique Missiakas

**Affiliations:** 1 Department of Microbiology, Howard Taylor Ricketts Laboratory, The University of Chicago, Lemont, Illinois, USA; 2 Department of Microbiology and Immunology, Faculty of Pharmacy, Sinai University, Arish, Egypt; The University of Kansas Medical Center, Kansas City, Kansas, USA

**Keywords:** *Staphylococcus aureus*, cross-wall, lipoteichoic acid synthase, gentiobiosyldiacylglycerol, signal peptidase, protein secretion, YSIRK motif

## Abstract

Septal membranes of *Staphylococcus aureus* serve as the site of secretion for precursors endowed with the YSIRK motif. Depletion of *ltaS*, a gene required for lipoteichoic acid (LTA) synthesis, results in the loss of restricted trafficking of YSIRK precursors to septal membranes. Here, we seek to understand the mechanism that ties LTA assembly and trafficking of YSIRK precursors. We confirm that catalytically inactive lipoteichoic acid synthase (LtaS)_T300A_ does not support YSIRK precursor trafficking to septa. We hypothesize that the enzyme’s reactants [gentiobiosyldiacylglycerol (Glc_2_-DAG) and phosphatidylglycerol (PG)] or products [LTA and diacylglycerol (DAG)], not LtaS, must drive this process. Indeed, we observe that septal secretion of the staphylococcal protein A YSIRK precursor is lost in *ypfP* and *ltaA* mutants that produce glycerophosphate polymers [poly(Gro-P)] without the Glc_2_-DAG lipid anchor. These mutants display longer poly(Gro-P) chains, implying enhanced PG consumption and DAG production. Our experiments also reveal that in the absence of Glc_2_-DAG, the processing of LtaS to the extracellular catalytic domain, eLtaS, is impaired. Conversely, LTA polymerization is delayed in a strain producing LtaS_S218P_, a variant processed more slowly than LtaS. We conclude that Glc_2_-DAG binding to the enzyme couples catalysis by LtaS and the physical release of eLtaS. We propose a model for the temporal and localized assembly of LTA into cross-walls. When LtaS is not processed in a timely manner, eLtaS no longer diffuses upon daughter cell splitting, LTA assembly continues, and the unique septal-lipid pool, PG over DAG ratio, is not established. This results in profound physiological changes in *S. aureus* cells, including the inability to restrict the secretion of YSIRK precursors at septal membranes.

**IMPORTANCE:**

In *Staphylococcus aureus*, peptidoglycan is assembled at the septum. Dedicated cell division proteins coordinate septal formation and the fission of daughter cells. Lipoteichoic acid (LTA) assembly and trafficking of preproteins with a YSIRK motif also occur at the septum. This begs the question as to whether cell division components also recruit these two pathways. This study shows that the processing of lipoteichoic acid synthase (LtaS) to extracellular LtaS by signal peptidase is regulated by gentiobiosyldiacylglycerol (Glc_2_-DAG), the priming substrate for LTA assembly. A model is proposed whereby a key substrate controls the temporal and spatial activity of an enzyme. In turn, this mechanism enables the establishment of a unique and transient lipid pool that defines septal membranes as a targeting site for the secretion of YSIRK preproteins.

## INTRODUCTION

The multilayer peptidoglycan of Gram-positive bacteria provides the mechanical strength to withstand the internal pressure of the cytosol. Peptidoglycan also represents a surface organelle for the attachment of polymers and surface proteins that define molecular interactions with the bacterial environment (1). Proteins attached to peptidoglycan are synthesized with specific topogenic sequences and require dedicated machinery for assembly, function, and release (1). In *Staphylococcus aureus,* the housekeeping sortase A enzyme recognizes the LPXTG motif within the C-terminal sorting signal of secreted surface proteins. Sortase A cuts this motif and covalently links substrate proteins to the staphylococcal pentaglycine cross-bridge of peptidoglycan (2–6). Some surface proteins are synthesized as precursors with an N-terminal cleavable signal sequence that carries the so-called YSIRK/GXXS (YSIRK) motif (7, 8). The YSIRK motif is found in streptococcal and staphylococcal species but not in the genera *Actinomyces*, *Bacillus*, *Clostridium*, and *Listeria* (9, 10). Staphylococci and streptococci have spherical and ovoid cell shapes and assemble peptidoglycan at, or near, septal membranes, a compartment between dividing cells designated as the cross-wall (11–13). In *S. aureus*, nascent cross-walls are split along a central axis to complete the cell cycle (1, 14). The divisome, with its core component FtsZ, directs the cell wall assembly machinery to the mid-cell to coordinate constriction events leading to daughter cell division and separation (15–17). Earlier work demonstrated that the YSIRK motif of precursor proteins, such as staphylococcal protein A (SpA), is not required for sortase-catalyzed anchoring to peptidoglycan (18). Instead, in streptococci and staphylococci, the YSIRK motif is necessary and sufficient to target precursors to septal membranes (7, 19). Immobilization by sortase made it possible to use fluorescence microscopy and visualize newly emerging precursors bearing a YSIRK motif (YSIRK precursors) at the cross-walls, while anchoring of non-YSIRK precursors (carrying a canonical signal sequence) was observed at polar peptidoglycan (7, 19). As a result, YSIRK precursors are readily distributed over roughly half of the bacterial surface following daughter cell separation (8, 20). A cross-linking approach identified SecA, lipoteichoic acid synthase (LtaS), penicillin-binding protein 2 (PBP2), and EzrA as candidate factors that could interact with SpA precursors in *S. aureus* (21). However, biochemical and genetic analyses revealed that divisome-associated PBP2 and EzrA do not play any role in SpA secretion (21). Thus, cross-linking of these proteins can be interpreted as a result of proximity rather than direct engagement with the cell division machinery (15, 21). The essential SecA protein was shown to bind both canonical and YSIRK-containing precursors, suggesting that SecA alone is not responsible for secretion at distinct sites (21). On the other hand, the depletion of *ltaS* that encodes the enzyme that assembles lipoteichoic acid (LTA) resulted in the indiscriminate secretion of SpA, which was no longer restricted to the cross-walls (21). Here, we aim to investigate how LtaS supports YSIRK targeting into septal compartments. We entertain two possibilities: (i) LtaS alone or in complex with other proteins recruits SecA-YSIRK precursor complexes, and (ii) alternatively, optimal targeting of YSIRK precursors requires the enzymatic activity of LtaS. Both scenarios assume that LTA assembly occurs at septal membranes, although a mechanism to recruit or limit LtaS activity at these sites has not been elucidated. In agreement with an earlier report (22), we find that bacteria producing the catalytically inactive LtaS protein (LtaS_T300A_) can no longer restrict SpA secretion into the cross-walls. Thus, we focus on the enzymatic activity of LtaS and ask whether depletion or accumulation of membrane lipids associated with LTA assembly may impact YSIRK precursor secretion. We use a genetic approach to deplete or alter the reactants and products of LtaS. We find that gentiobiosyldiacylglycerol (Glc_2_-DAG), the priming substrate of LtaS, plays a key role in restricting LTA assembly at septal membranes. We propose that limiting LTA assembly at septal membranes establishes a unique lipid environment necessary for the recruitment of YSIRK precursors.

## RESULTS

### LTA synthesis, not LtaS protein, is required for the secretion of YSIRK precursors into the cross-wall compartment

**Fig 1:**
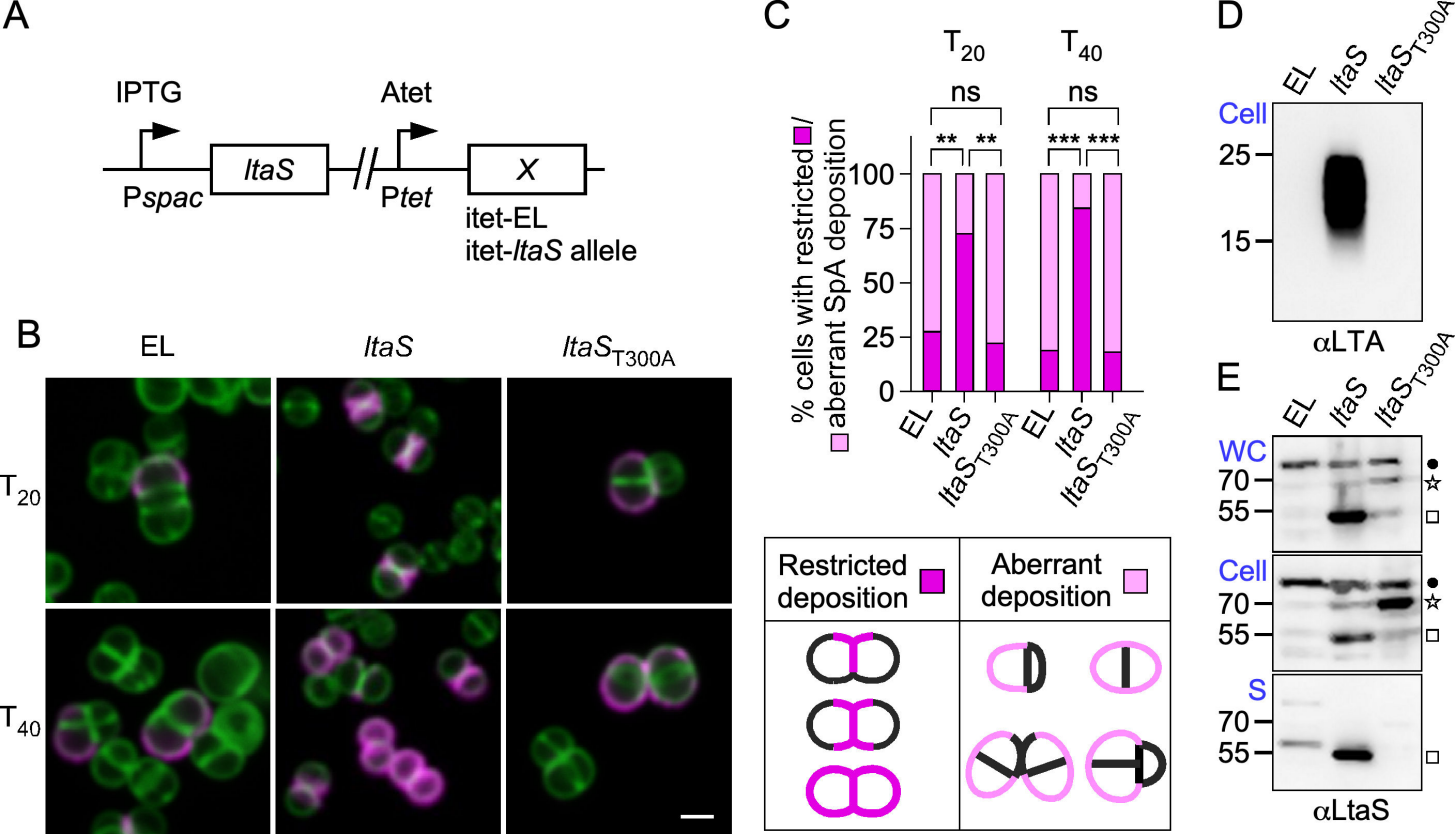
LTA synthesis, not LtaS protein, is required for the secretion of SpA precursors into the cross-wall compartment. (**A**) Diagram depicting the genetic makeup of strains. Pairwise expression of chromosomally encoded *ltaS* genes. The P*_spac_* promoter controls the expression of the wild-type *ltaS* gene, while locus *X* carries *ltaS* variants under the P*_tet_* promoter; EL indicates that the locus was left empty. (**B and C**) Distribution of SpA in the cell wall envelope of staphylococci. *S. aureus* bacteria were treated with trypsin to remove surface proteins and allowed to recover for 20 and 40 min (*T*_20_/*T*_40_) before fluorescence microscopy using BODIPY FL-vancomycin (green) to visualize the cell wall and SpA-specific antibodies followed by secondary goat anti-human conjugated to Alexa Fluor 594 (magenta). Two-dimensional two-color images were acquired using a Stellaris 8 confocal microscope, and images were processed using ImageJ to change the red channel into magenta. Scale bars = 2 µm. Representative images are shown in panel **B**. The SpA signal was calculated from at least two independent experiments as the percentage of cells with restricted (normal) or aberrant SpA deposition, as shown in drawings, with respect to the total number of cells counted. Data were analyzed using two-way ANOVA with Tukey’s multiple comparison test (**P = 0.0058 and 0.0033, respectively; ***P < 0.0001). (**D and E**) LTA (**D**) and LtaS (**E**) production was examined by immunoblotting samples from whole cultures (WC), washed cells (Cell), or the extracellular supernatant (S) of bacterial strains grown for 2 h in the absence of IPTG followed by 1 h in the presence of Atet. Blots were analyzed with antibodies against LTA (αLTA) in panel D or against LtaS (αLtaS) in panel E. The star and square identify the LtaS precursor (MW 70 kDa) and mature protein (MW 55 kDa), respectively, while the dot identifies an unknown protein cross-reactive with αLtaS (see Fig. S1 for details). The sizes of the MW markers are shown to the left.

**Fig 2:**
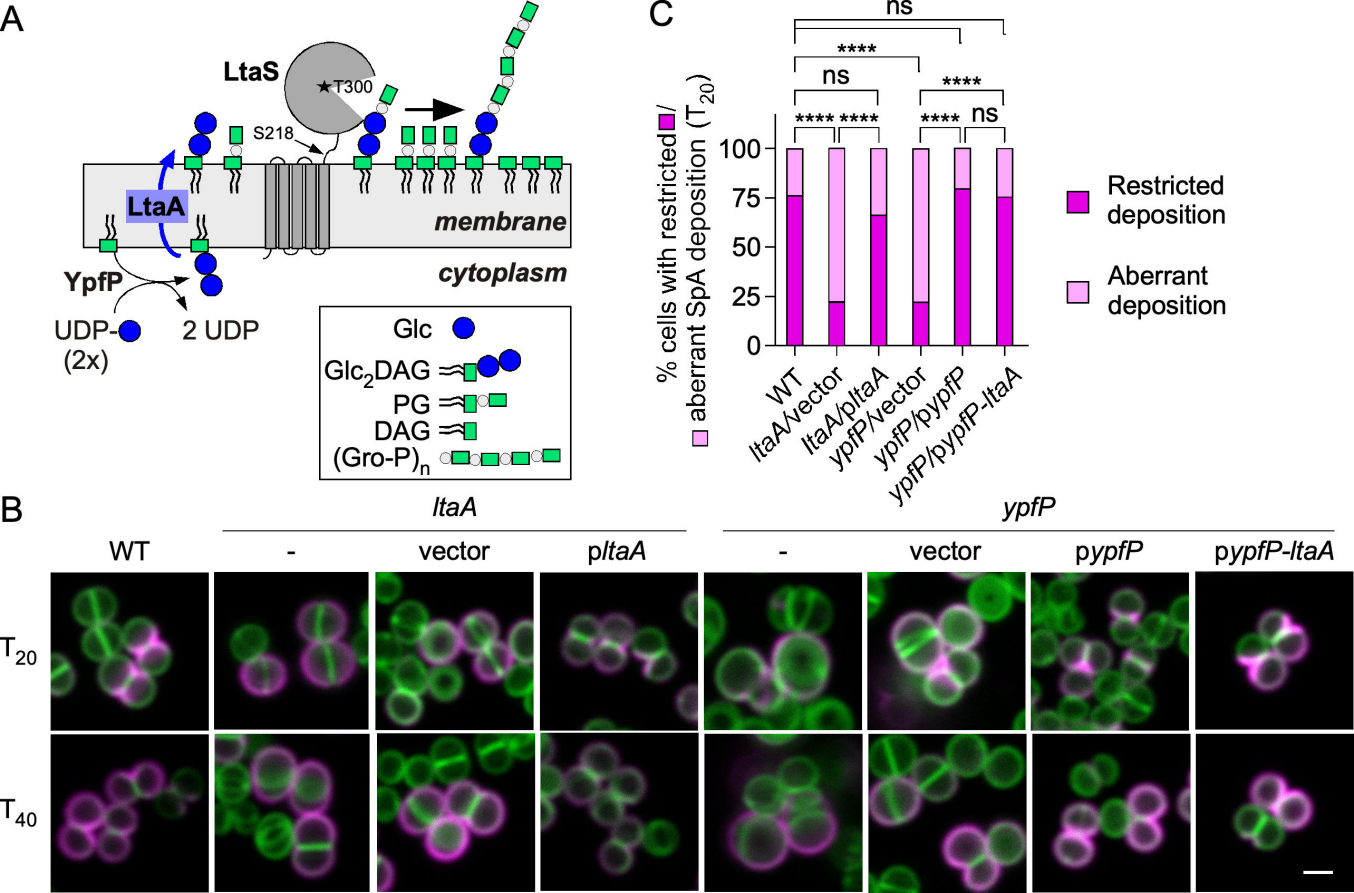
Depleting the glycolipid anchor of LTA affects septal secretion of SpA. (**A**) Schematic representation of the LTA assembly pathway. YpfP synthesizes the last step of the Glc_2_-DAG glycolipid anchor that is flipped across the bilayer by LtaA and serves to initiate the LtaS-mediated transfer of glycerophosphate (Gro-P) subunits from phosphatidylglycerol (PG). This reaction leads to the release of DAG. S218 and T300 represent the processing and active sites of LtaS, respectively. (**B and C**) Distribution of SpA in the cell wall envelope of staphylococci visualized by microscopy (**B**) and quantified for septal trafficking (**C**). *S. aureus* wild-type (WT), *ltaA*, and *ypfP* mutants and their complemented strains were viewed as described in Fig. 1 following trypsin removal of surface proteins, and bacteria were allowed to recover for 20 and 40 min (*T*_20_/*T*_40_). Scale bars = 2 µm. (**C**) Septal versus aberrant SpA trafficking was quantified at *T*_20_ from two independent experiments as described in Fig. 1. Data were analyzed using one-way ANOVA with Tukey’s multiple comparison test (****P < 0.0001).

LtaS-mediated synthesis of LTA is essential for *S. aureus* growth and cell division (23). Earlier work established a genetic system to study the activity of LtaS *in vivo* (23, 24). Briefly, the *S. aureus* strain ANG499 (RN4220 P*_spac_-ltaS*) expresses chromosomally encoded *ltaS* under the tightly controlled isopropyl thiogalactoside (IPTG)-inducible promoter P*_spac_* (Fig. 1A). A second locus in strain ANG499 carries the anhydrotetracycline (Atet)-inducible promoter (P*_tet_*) that is used to drive the expression of either the wild-type or mutant LtaS, such as the catalytically inactive LtaS_T300A_ variant (with threonine 300 replaced with alanine), or is left empty [empty locus (EL)] with no gene inserted at this site (Fig. 1A) (23, 24). In the absence of inducers, the strains stop producing LTA and cease to grow. The addition of Atet rescues these defects in the merodiploid strain producing wild-type *ltaS* (P*_spac_-ltaS,*P*_tet_-ltaS*) but not *ltaS*_T300A_ (P*_spac_-ltaS,*P*_tet_-ltaS*_T300A_) or when the locus is left empty (EL, P*_spac_-ltaS,*P*_tet_-*EL). Here, the three strains, simply referred to as EL, *ltaS*, and *ltaS*_T300A_ (Fig. 1), were grown overnight in the presence of IPTG (permissive condition). The next day, cultures were inoculated in medium without IPTG for 2 h to deplete LtaS before the addition of Atet, and the deposition of SpA in the envelope was observed using immunofluorescence microscopy following a well-established protocol (7, 21). Briefly, washed cells were treated with trypsin to shave all proteins from the bacterial surface and incubated for 20 min (*T*_20_) and 40 min (*T*_40_) in the presence of a trypsin inhibitor; *T*_20_ and *T*_40_ represent roughly one and two cell division cycles, respectively (7, 21). Following fixation, SpA was stained with a monoclonal antibody (αSpA) and Alexa Fluor 594-conjugated secondary IgG (ImageJ was used to swap red to magenta in all the images). BODIPY FL-vancomycin (green) was used to stain peptidoglycan. As expected, in cells expressing wild-type LtaS, SpA molecules appeared along the Y or X shapes of splitting cross-walls (septal location) (Fig. 1B and C). Cells depleted of wild-type LtaS (EL) have aberrant sizes and septa (23, 24); these cells do not divide efficiently but continue to produce SpA, with staining observed randomly around the envelope at both *T*_20_ and *T*_40_ (Fig. 1B and C), supporting the notion that SpA secretion is no longer synchronized with cell division or restricted to cell septa. Similarly, the restricted septal deposition of SpA was lost in cells expressing *ltaS*_T300A_ that also failed to divide properly (Fig. 1B and C). These observations mirror results obtained by Zhang et al. (22) using a set of *ltaS* mutant strains carrying an extragenic suppressor allele that restores growth (25). The lack of LTA production and the presence of the catalytically inactive LtaS_T300A_ protein were documented by immunoblotting extracts of *S. aureus* cells and cultures, respectively (Fig. 1D). The latter analysis, comparing whole cultures (WC) versus cells (Cell) separated from the culture supernatant (S), revealed that LtaS_T300A_ accumulates as a sedimentable 70-kDa species (Fig. 1E). Instead, LtaS is processed to the 55-kDa species and found in the culture supernatant (Fig. 1E). This agrees with previous reports demonstrating that the extracellular catalytic domain of LtaS, 55-kDa eLtaS, is cleaved from the polytopic N-terminal transmembrane domain (Fig. 2A) (23, 24, 26). The 70-kDa species represents full-length, membrane-bound LtaS before processing by signal peptidase B (SpsB) at serine 218 (S218) (Fig. 2A) (26). A band slightly above the 70-kDa marker was ruled out as a non-specific cross-reactive species, as noted by others (26) (Fig. S1). Thus, similar to Zhang et al. (22), we observe that the expression of *ltaS*_T300A_ fails to restrict the secretion of SpA precursors at the septum, suggesting that the LtaS catalyst, not the LtaS protein, plays an important role in targeting YSIRK precursors to the cross-walls. Surprisingly, we observe that catalytically inactive LtaS_T300A_ is not processed to a 55-kDa species, suggesting that catalysis and processing are coupled events.

### LtaS processing and septal secretion of SpA are altered by the depletion of Glc_2_-DAG

**Fig 3:**
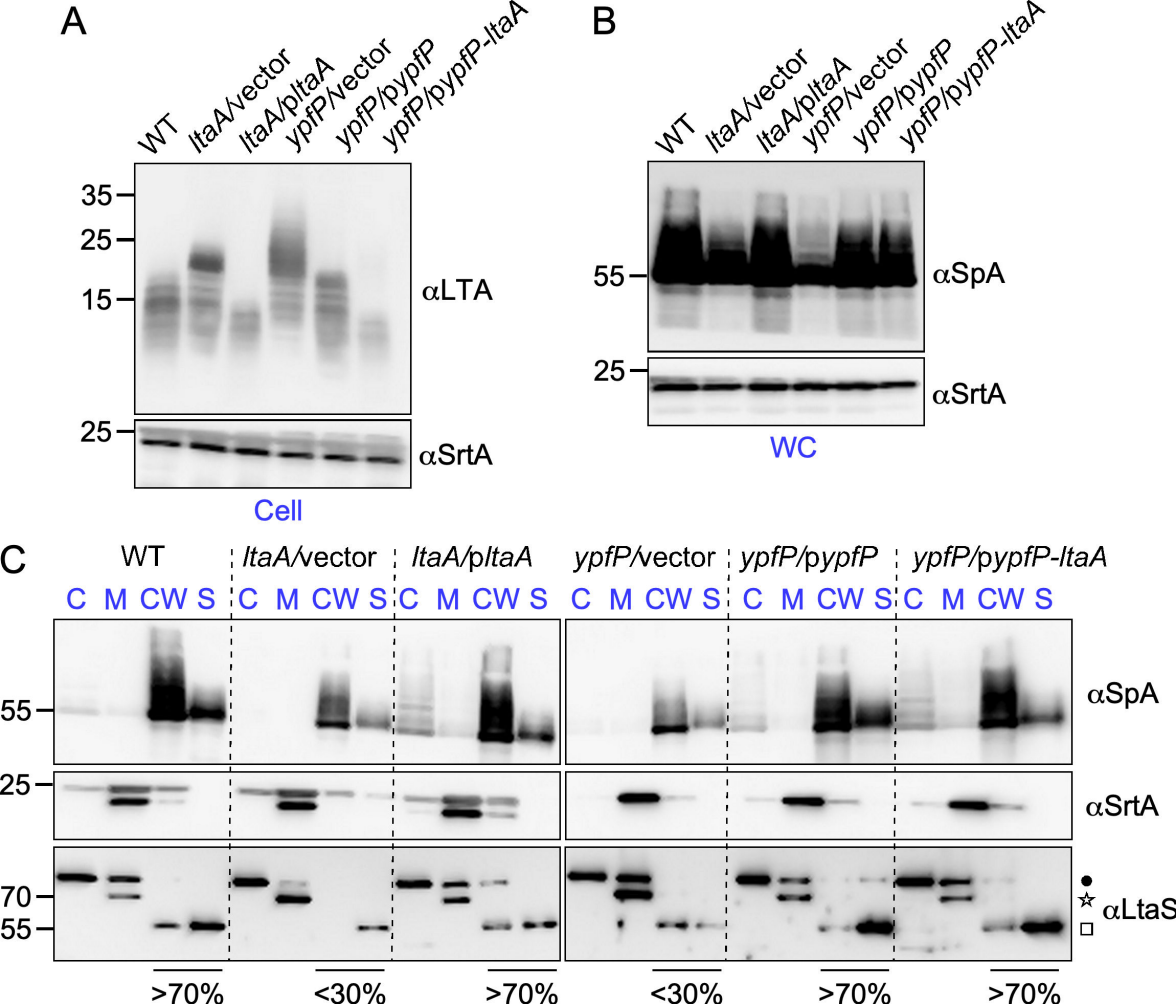
Depleting the glycolipid anchor of LTA affects LtaS processing. (**A to C**) Production of LTA, SpA, and LtaS was examined in *S. aureus* wild-type (WT), *ltaA*, and *ypfP* mutants and their complemented strains. Extracts were prepared using washed lysed cells (Cell; panel A), whole cultures (WC; panel B), and subcellular fractions (C, cytoplasm; M, membrane; CW, cell wall; S, culture supernatant; panel C). Samples were separated on gels and transferred to membranes for immune detection with antibodies against LTA (αLTA), LtaS (αLtaS), and sortase A (αSrtA) as loading controls. The star, square, and dot identify the LtaS precursor (MW 70 kDa), the mature protein (MW 55 kDa), and an unknown protein cross-reactive species as described in Fig. 1. The fraction of processed eLtaS present in combined CW and S lanes is indicated under the blots as compared to total LtaS immune reactive signals (100%) scanned in all four lanes (C, M, CW, and S). The sizes of the MW markers are shown to the left of the blots.

*S. aureus* LTA is composed of 1,3-polyglycerol phosphate [poly(Gro-P)] linked to the C-6 of the nonreducing glycosyl of the glycolipid anchor, gentiobiosyldiacylglycerol [Glc(β1–6)Glc(β 1–3)-diacylglycerol] also named diglucosyldiacylglycerol, Glc_2_-DAG, for short (27). LtaS polymerizes poly-glycerol-P chains of 15–50 units, poly(Gro-P), onto Glc_2_-DAG (i.e., LTA) through the stepwise addition of *sn*-glycerol-1-phosphate obtained from the head group of the membrane lipid phosphatidylglycerol (PG) (27–30) (Fig. 2A). Thus, LtaS uses two substrates, Glc_2_-DAG and PG, to generate LTA and the by-product diacylglycerol (DAG) (Fig. 2A) (29). In the absence of active LtaS, the aberrant content of PG, Glc_2_-DAG, DAG, or poly(Gro-P) could be responsible for the loss of SpA secretion into the cross-walls. To test this hypothesis, we focused first on the well-characterized pathway of Glc_2_-DAG synthesis that takes place in the bacterial cytoplasm through the sequential action of PgcA (phosphoglucomutase), GtaB (UTP-Glc-1P uridyltransferase), and YpfP (diacylglycerol glucosyltransferase) (30, 31). Glc_2_-DAG, the final product of YpfP, is located on the *cis* side of the membrane until its translocation to the *trans* side by LtaA (Fig. 2A). We use *ypfP* and *ltaA* mutants to examine the impact of Glc_2_-DAG on SpA secretion. As reported earlier, cells of *ypfP* and *ltaA* mutants appeared slightly larger than wild-type cells and displayed morphological defects (Fig. 2B) (32). In these mutants, immobilization of SpA was no longer restricted to the cross-walls of dividing cells (Fig. 2B and C; Fig. S2). *ypfP* is found in an operon upstream of *ltaA*, and complementation studies were undertaken using plasmids with constitutive expression of each single gene (p*ypfP* or p*ltaA*) or both genes (p*ypfP-ltaA*). Both the p*ypfP* and p*ypfP-ltaA* plasmids restored SpA localization to the cross-walls at *T*_20_, ruling out any polar effect of *ypfP* gene disruption. Similarly, plasmid p*ltaA* complemented both the morphological defects and secretion into the cross-walls (Fig. 2B and C; Fig. S2), even though the overall SpA staining was not as bright in the complemented strain (*ltaA*/p*ltaA*) (Fig. S2). Together, the data suggest that SpA targeting to cross-walls is impeded in *ypfP* cells that lack the glycolipid anchor Glc_2_-DAG and in *ltaA* mutant cells that fail to translocate Glc_2_-DAG to the *trans* side of the plasma membrane. Chains of poly(Gro-P) are longer in bacteria lacking *ypfP* and tethered to DAG (30, 32). In *ltaA* mutants, most poly(Gro-P) is also tethered to DAG, albeit a small amount is anchored to Glc_2_-DAG (possibly because of redundant activity by an LtaA homolog) (30). The altered mobility of poly(Gro-P) species was confirmed here by separating *ypfP* and *ltaA* extracts by electrophoresis followed by western blotting (Fig. 3A). Plasmid complementation restored the length of LTA polymers, albeit complementation with plasmids bearing *ltaA* resulted in polymers slightly shorter than those in wild-type bacteria (Fig. 3A). Western blots of whole bacterial cultures (WC) revealed that *ypfP* and *ltaA* mutants produce reduced amounts of SpA, but this defect was restored upon plasmid complementation (Fig. 3B). Fractionation of bacterial cultures to separate the cytoplasm (C), membrane (M), cell wall (CW), and culture supernatant (S) was also used to examine the fate of LtaS (Fig. 3C). As expected, 70-kDa LtaS sedimented with the membrane, but in wild-type bacteria, the majority of the protein (>70%) was processed to eLtaS (55 kDa) and observed in the cell wall and mostly the supernatant fraction (Fig. 3C). The opposite was observed in *ypfP* and *ltaA* cells (Fig. 3C). Most LtaS (>70%) remained unprocessed and sedimented with the membrane fraction (Fig. 3C). Thus, in *ypfP* and *ltaA* mutants, both the chemical nature of LTA (composition and length) and the processing of LtaS are altered.

### Do other membrane lipids impact LtaS processing and septal secretion of SpA?

**Fig 4:**
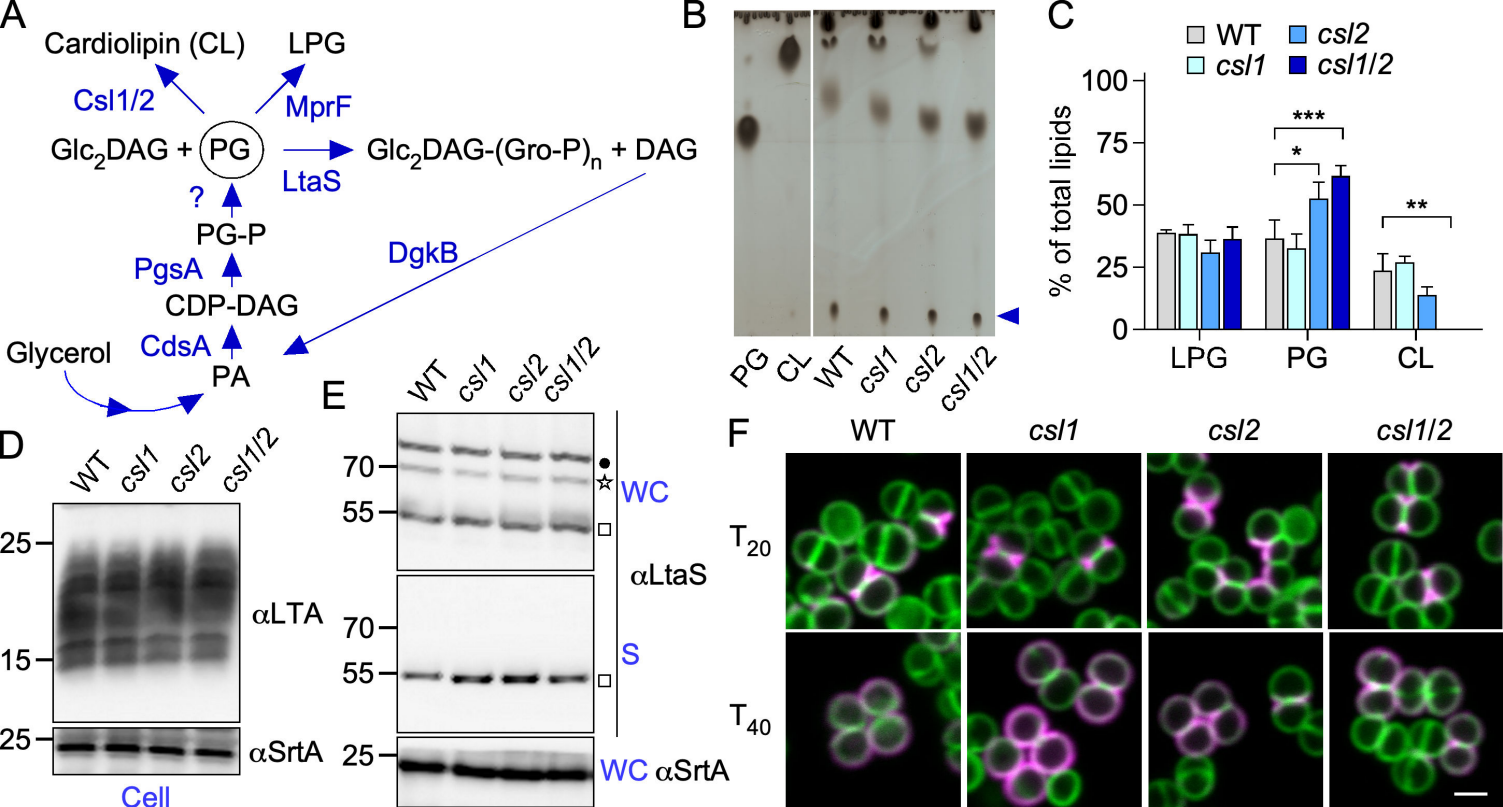
Altering CL levels does not impact septal secretion of SpA. (**A**) Schematic representation of the pathways leading to the production and consumption of PG in *S. aureus*. (**B**) Thin-layer chromatography (TLC) of lipids extracted from *cls1*, *cls2*, and *cls1/2 S. aureus* mutants. One representative TLC plate of three independent experiments is shown. Migration of PG and CL standards was used to identify lipids. The blue arrow points to the migration of LPG. (**C**) Quantification of lipids identified by TLC. SEM was derived from three independent experiments, and data were analyzed with two-way ANOVA (Dunnett’s multiple comparison test; ***P < 0.001; **P < 0.01; *P < 0.05). (**D and E**) Immunoblots of bacterial extracts prepared from washed lysed cells (Cell, panel D) and supernatant (S) or whole cultures (WC) (panel E). Blots were analyzed with antibodies against LtaS (αLtaS), sortase A (αSrtA), and LTA (αLTA). The star, square, and dot identify the LtaS precursor (MW 70 kDa), the mature protein (MW 55 kDa), and an unknown protein cross-reactive with αLtaS, respectively. (**F**) Distribution of SpA in the cell wall envelope of staphylococci. *S. aureus* bacteria were viewed as described in Fig. 1. Scale bars = 2 µm.

In addition to Glc_2_-DAG, LtaS uses PG to generate LTA and the by-product DAG (Fig. 4A). In *S. aureus*, PG is also converted to lysyl phosphatidylglycerol (LPG) and cardiolipin (CL) (33) (Fig. 4A). PG, LPG, and CL constitute 38%–76%, 14%–38%, and 5%–30% of the total phospholipids, respectively (34). In bacteria, PG is synthesized from phosphatidic acid (PA) upon conversion to CDP-diacylglycerol (CDP-DAG) by the phosphatidate cytidylyltransferase enzyme, CdsA (33). Next, PgsA transfers CDP-DAG onto *sn*-glycerol-3-phosphate to generate CMP, and 3(3-*sn*-phosphatidyl)-*sn*-glycerol-1-phosphate (PG-P) and redundant phosphatases (Pgp enzymes) catalyze the dephosphorylation of PG-P to PG (33). In *S. aureus*, the *cdsA* and *pgsA* genes are thought to be essential, and Pgp-like phosphatases have not been identified (33, 35). A genetic approach to alter the flow of PG is thus challenging. However, the genetic determinants for CL synthesis (*cls1* and *cls2*) are not required for *S. aureus* growth (36, 37). Thus, we sought to grossly alter the pool of membrane phospholipids by mutating *csl1* and *csl2*. Lipids extracted from the wild-type and mutant bacteria were separated by thin-layer chromatography (TLC) (Fig. 4B). As reported by others, the lack of *cls1* did not result in any major changes (36, 37), but the lack of *csl2* either alone or combined with *cls1* resulted in increased PG (Fig. 4B and C). The CL pool was completely depleted in the absence of both the *cls1* and *cls2* genes, but the levels of LPG were unchanged (Fig. 4B and C). Despite these major changes, LTA assembly (Fig. 4D) and LtaS processing to eLtaS were unaffected (Fig. 4E). These results point to the notion that PG is not limiting in wild-type bacteria, and augmenting its concentration has no impact on LTA assembly. Similarly, neither increased PG nor lack of CL altered SpA targeting to the cross-walls (Fig. 4F).

### LTA synthesis is coupled to LtaS processing

**Fig 5:**
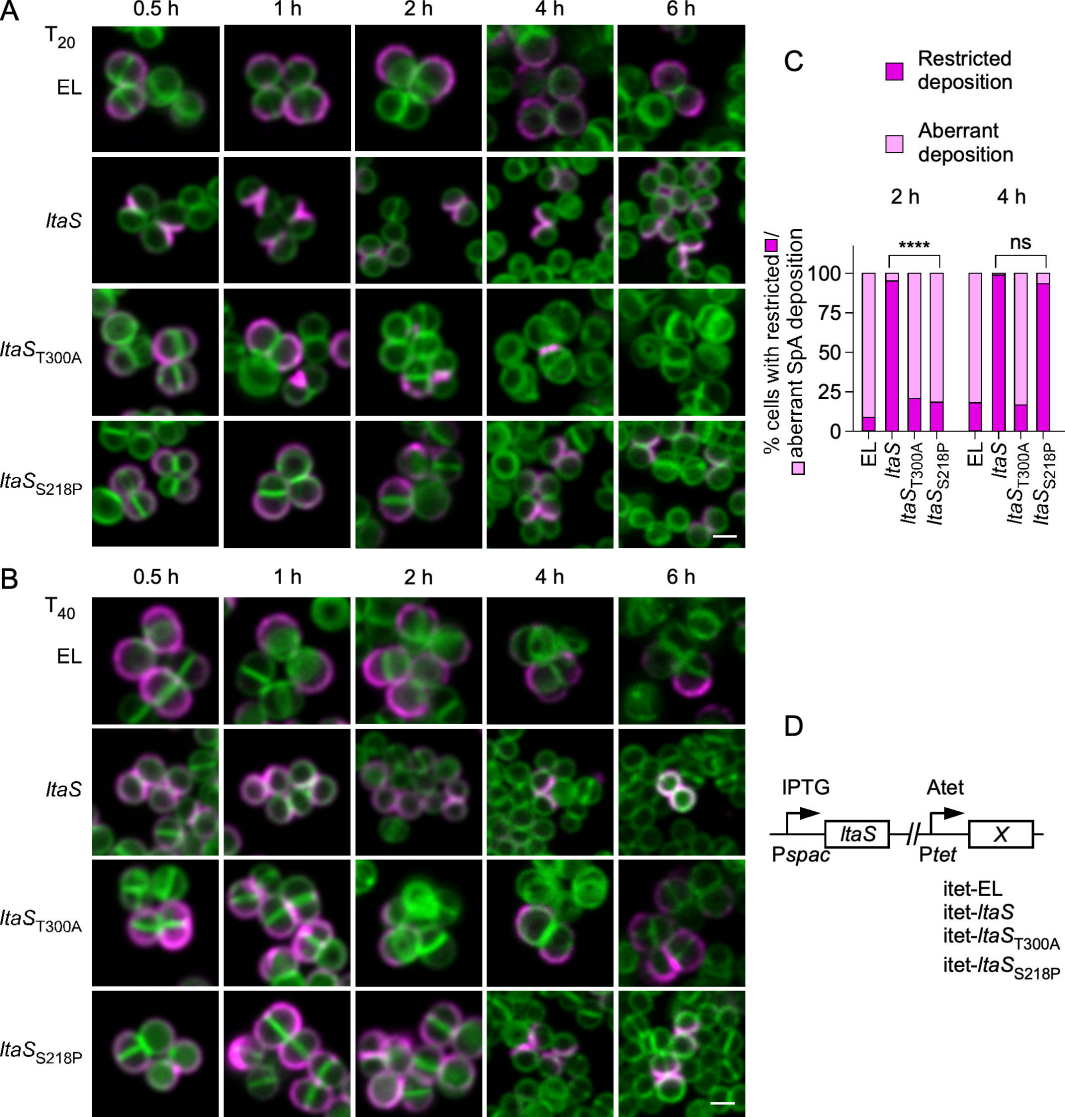
*ltaS* alleles that impact septal secretion of SpA. Bacterial cultures were grown overnight under permissive conditions (+ IPTG) and subsequently subcultured in fresh medium without IPTG for 2 h before the addition of Atet. (A and B) Samples were collected at 0.5, 1, 2, 4, and 6 h post-Atet induction. The distribution of SpA was examined at each of these time points by microscopy following trypsin removal of surface proteins, fixation, and recovery for 20 min (*T*_20_, panel A) and 40 min (*T*_40_, panel B). Scale bars = 2 µm. *ltaS* merodiploid strains used for this experiment are as described in Fig. 1 and express *ltaS* alleles under the P*tet* promoter; EL indicates that the locus was left empty. (**C**) Quantification of SpA display was performed as described in Fig. 1 from three independent experiments. Data were analyzed using two-way ANOVA with Tukey’s multiple comparison test (****P < 0.0001). (**D**) Diagram depicting the genetic makeup of strains. The P*_spac_* promoter controls the expression of the wild-type *ltaS* gene, while locus *X* carries *ltaS* variants under the P*_tet_* promoter; EL indicates that the locus was left empty.

**Fig 6:**
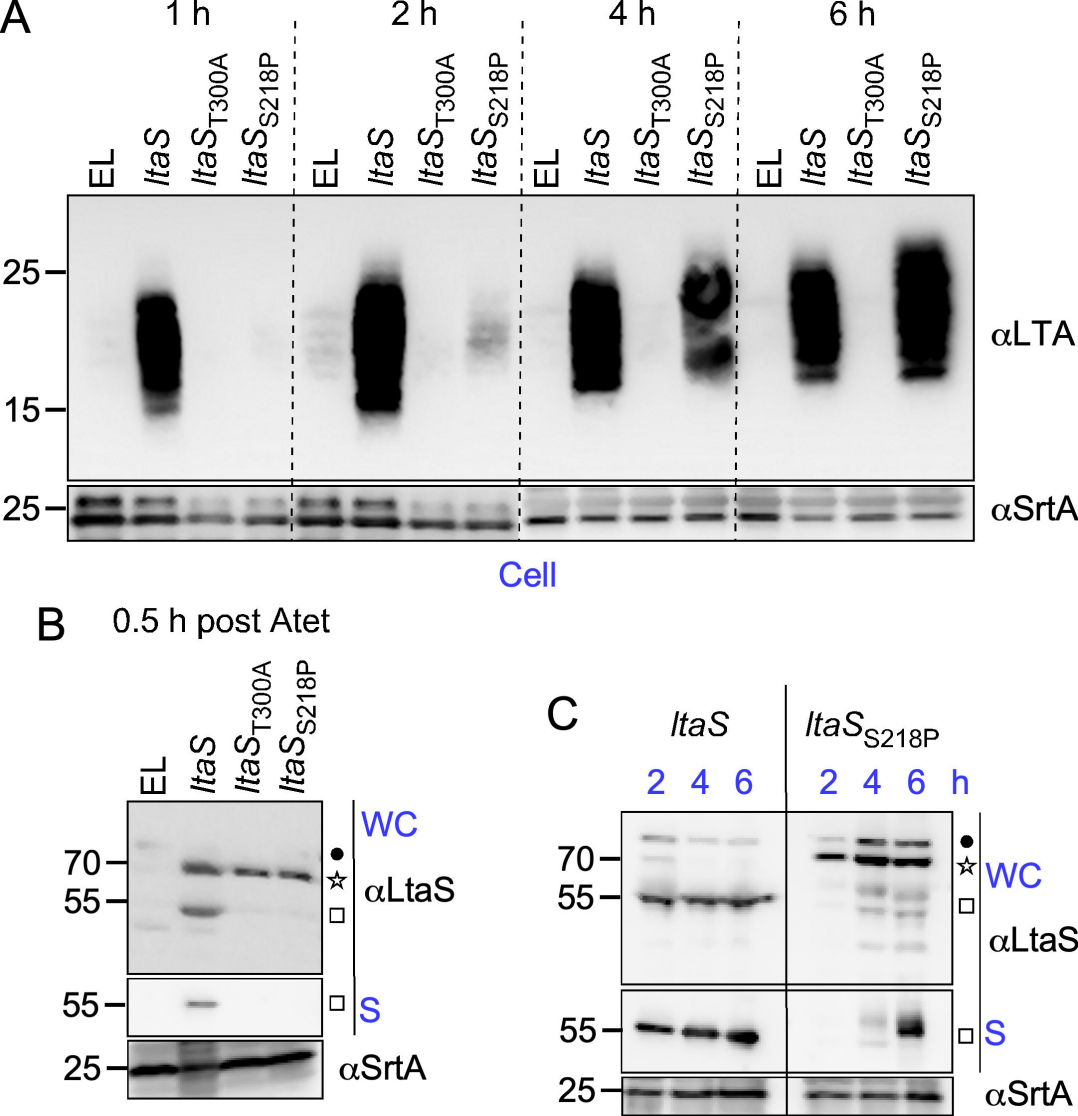
LtaS processing is coupled to its polymerase activity. (**A to C**) Production of LTA and LtaS was examined by western blotting. Bacterial cultures were grown as described in Fig. 5. Overnight cultures were grown under permissive conditions (+ IPTG) and subsequently subcultured in fresh medium without IPTG for 2 h before the addition of Atet for 0.5, 1, 2, 4, and 6 h as indicated above. Immune signals for LTA and LtaS were probed in extracts from washed cells (Cells), whole cultures (WC), or supernatants (S) as indicated with antibodies as described in Fig. 1. Sortase A (SrtA) was used as a loading control. The star, square, and dot identify the LtaS precursor (MW 70 kDa), the mature protein (MW 55 kDa), and an unknown protein cross-reactive with αLtaS, respectively. Extracts were prepared using merodiploid strains expressing the three *ltaS* alleles under the P*tet* promoter, as depicted in Fig. 5D. EL indicates that the locus was left empty.

Since catalytically inactive LtaS_T300A_ is not processed and optimal LtaS processing requires Glc_2_-DAG, it seems reasonable to assume that these two processes are coupled. Wörmann et al. showed earlier that SpsB cleaves LtaS between alanine 217 and serine 218 (26). An attempt to eliminate the SpsB site by substituting serine 218 with proline (LtaS_S218P_) resulted in alternate, less effective processing between residues valine 191 and lysine 192 (26). We exploited this construct to perform a kinetic study and explore the relationship between LtaS processing, LTA synthesis, and SpA secretion. Following the same strategy described in Fig. 1A, *ltaS*_S218P_ was placed under the P*_tet_* promoter (P*_spac_-ltaS,*P*_tet_-ltaS*_S218P_) of *S. aureus* ANG499 (24). Herein, this strain is referred to as *ltaS*_S218P_ and is used along the merodiploid ANG499 variants, *ltaS* (P*_spac_-ltaS,*P*_tet_-ltaS*), *ltaS*_T300A_ (P*_spac_-ltaS,*P*_tet_-ltaS_T300A_*), and EL (P*_spac_-ltaS,*P*_tet_-*EL) (Fig. 5D). Bacterial cultures were grown overnight under permissive conditions (+ IPTG). The medium was then replaced with fresh tryptic soy broth (TSB), and subcultures were grown without any inducer for 2 h to shut down P*_spac_-ltaS* expression before the addition of Atet. Sample aliquots were collected 0.5, 1, 2, 4, and 6 h post-Atet induction to examine SpA secretion (*T*_20_/*T*_40_) using microscopy (Fig. 5A through C), as well as to evaluate the production of LTA (Fig. 6A) and LtaS (Fig. 6B and C). As expected, SpA secretion was not restricted to septal membranes in EL cells that cannot express *ltaS* or in cells expressing *ltaS*_T300A_ (Fig. 5A through C). Cells carrying the wild-type *ltaS* gene displayed the typical staining of SpA at splitting cross-walls at *T*_20_, with an even SpA distribution at *T*_40_, after the addition of the trypsin inhibitor. Importantly, secretion into the cross-walls was observed at the shortest (0.5 h) incubation time with Atet (Fig. 5A through C). In comparison, cross-wall deposition of SpA was delayed up to 4 h post-Atet treatment in *ltaS*_S218P_ cells (Fig. 5A through C). This delay correlated with a delayed production of LTA in *ltaS*_S218P_ cells (Fig. 6A). In comparison, LTA products were detectable within 1 h post-Atet induction in extracts of *ltaS* cells and absent in control extracts (EL and *ltaS*_T300A_; Fig. 6A). The delayed LTA production was not due to the lack of enzyme synthesis, as western blotting of whole bacterial cultures (WC) demonstrated similar levels of LtaS, LtaS_ST300A_, and LtaS_S218P_ proteins following the addition of Atet for 0.5 h (Fig. 6B). However, the processing and release of eLtaS (55-kDa species) was only observed in WC/S extracts prepared from merodiploid P*_spac_-ltaS,*P*_tet_-ltaS* bacteria (Fig. 6B). Samples were also collected 2, 4, and 6 h post-Atet induction (Fig. 6C), i.e*.,* before (2 h) and after (4 and 6 h) septal targeting (Fig. 5) and LTA production (Fig. 6A). Western blotting revealed that 2 h following Atet induction, wild-type LtaS was mostly processed to eLtaS and found in the culture supernatant (S), while 70-kDa LtaS_S218P_ accumulated in the whole culture extracts (Fig. 6C). eLtaS_S218P_ was not detected in the supernatant fraction until at least 4 h post-Atet induction (Fig. 6C). We surmise that unprocessed LtaS_S218P_ produces LTA less effectively *in vivo*; the appearance of LTA was delayed by more than 2 h in bacteria expressing *ltaS*_S218P_ as compared to *ltaS*, mirroring the delay in both LtaS processing and SpA trafficking to the cross-walls.

## DISCUSSION

In *S. aureus*, cytokinesis is initiated by FtsZ, a tubulin homolog with GTP-dependent polymerization activity (15, 17). FtsZ assembles into filaments in the nucleoid-free region of the cell to form a ring-like structure (Z ring). The Z ring serves as a scaffold for the sequential recruitment of conserved proteins that form the so-called divisome to coordinate cell division and new peptidoglycan synthesis by PBP1–4, RodA, and FtsW (15, 17). After assembly of the divisome, the Z ring constricts, promoting invagination of the membrane accompanied by septal peptidoglycan synthesis. This invagination process divides the original cell into two equally sized daughter cells, with approximately one-third of the new cell surface originating from septa (38). The cross-walls, the site of septal peptidoglycan assembly, represent a closed compartment that is not accessible to large extracellular factors such as the hydrolytic enzyme, lysostaphin, and antibodies. The incorporation of serine in septal peptidoglycan naturally increases resistance toward lysostaphin (39). Thus, immune labeling of newly secreted proteins with a YSIRK motif such as SpA can only be achieved as the cross-walls are split. Similarly, immune labeling of LTA and WTA (wall teichoic acid) fails to reveal their presence at the septum (22, 40), although enzymes for the assembly of both anionic glycopolymers have been shown to be septally located (41, 42).

LTA and WTA are important molecules for *S. aureus*. Strains lacking teichoic acids (TAs) exhibit morphological abnormalities, thickened peptidoglycan, increased cell size, and defects in septal positioning and number (42, 43). WTA is linked to peptidoglycan and exposed on the cell surface, while LTA is tethered to the membrane and remains buried under the peptidoglycan mesh, where it remains inaccessible to labeling with antibodies (42, 43). A complex set of interactions has been noted between WTA and cell division. The cross-linking enzymes PBP4 and FmtA no longer accumulate at the division septum of bacteria lacking WTA (*tagO* mutants) (41, 44). TagG, the WTA export protein, interacts with GpsB, a modulator of FtsZ (45), while DivIC, a transmembrane protein of the late divisome, interacts preferentially with WTA-modified peptidoglycan (46). WTA is also thought to shield the underlying peptidoglycan from the major autolysin, Atl (43). Presumably, newly synthesized WTA that lacks substitutions (immature WTA) can influence septal cross-linking (41, 44), while mature, modified WTA on the cell surface restricts degradation by Atl (40).

LTA assembly has also been proposed to occur at the cross-walls, although a mechanism for recruitment has not been revealed (42). Fluorescent reporters of YpfP and LtaA have been observed all around the bacterial membrane, while an LtaS reporter was found to accumulate predominantly at the septum (42). Here, while examining the targeting of SpA to the cross-walls, we stumbled upon a mechanism that may account for LTA assembly at septal membranes without a requirement for direct interactions with divisome proteins. We propose a model whereby Glc_2_-DAG, the priming substrate of LtaS, triggers the processing of the enzyme governing the timed and spatial assembly of LTA. In theory, LtaS could insert anywhere in the membrane, but its engagement with Glc_2_-DAG triggers processing by SpsB, releasing the catalytic eLtaS fragment. When the enzyme inserts in septal membranes, eLtaS remains trapped in the cross-walls of dividing cells and continues to assemble poly(Gro-P) until cell splitting results in the release of eLtaS and irreversibly shuts down LTA polymerization. This model does not require a mechanism for re-localization of LtaS following cell division, as would be the case for membrane proteins of the WTA assembly pathway. We presume that the transmembrane domain of LtaS either carries a different function or is rapidly degraded. In support of our model, we observe that a catalytically inactive LtaS_T300A_ variant is not processed by SpsB. Such defective processing has been observed for other catalytically inactive variants (24). However, what happens when the SpsB cleavable site of LtaS is mutated? Such a variant, LtaS_S218P_, was generated and found to be cleaved at an alternate site (26). Genetic engineering permitted the sequential expression of wild-type and variant *ltaS* genes. By switching off the production of wild-type LtaS and inducing the expression of *ltaS*_S218P_ or *ltaS* at an alternate site, it was possible to interrogate the fate of the enzymes and LTA products. Western blotting revealed that LtaS_S218P_ processing is ineffective and delayed by roughly 2 h as compared to LtaS. Similarly, LTA production lagged by 2 h, and SpA targeting into the cross-walls was not observed until the production of LTA. Our model is in agreement with biochemical experiments examining the catalytic activity of LtaS reconstituted in proteoliposomes (47). These experiments revealed that Glc_2_-DAG, not PG, is the preferred starter unit of LtaS and may remain associated with LtaS to displace the growing Gro-P polymer and control the length of LTA molecules (47). Here, we suggest that LtaS interaction with Glc_2_-DAG is coupled with cleavage by SpsB. While cleaved eLtaS may remain associated with the transmembrane domain holding the substrates and products, SpsB processing ensures the irreversible dissociation of the catalytic domain upon cell splitting. Thus, while reducing the pool of Glc_2_-DAG (*ltaA* mutant) results in longer LTA (30, 32), increasing the pool of Glc_2_-DAG (plasmid overexpression of *ltaA*) results in shorter LTA polymers. Consequently, increased Glc_2_-DAG accelerates both the release of growing chains from the enzyme and the irreversible separation of eLtaS from the membrane. Together, these observations are in agreement with the notion that the expression of the transmembrane and extracellular domains of LtaS from two different plasmids would fail to reconstitute LtaS activity (26). Our conclusion differs slightly with respect to the role of LtaS processing, which is not merely to inactivate the enzyme but rather to control the timely and localized synthesis of LTA.

However, why should LTA assembly correlate with the recruitment of YSIRK precursors at septal membranes? Elongation of poly(Gro-P) leads to consumption of PG and production of DAG exclusively at septal membranes. We propose that disrupting LTA assembly alters the pool of both lipids, which ultimately results in the loss of SpA targeting to septal membranes. It is interesting that while DAG is a precursor in the synthesis of Glc_2_-DAG, a pathway to produce DAG in the absence of LTA assembly has not been identified in *S. aureus* (29, 48). DAG is recycled by DgkB (diacylglycerol kinase B) to PA (28, 49, 50) (Fig. 4A). *dgkB* expression is essential unless LTA synthesis is blocked, in which case DAG recycling and *dgkB* expression are obsolete for growth (49). Similarly, DAG is also a by-product in *Escherichia coli* and is used for the transfer of Gro-P from PG to form the periplasmic osmoregulant membrane-derived oligosaccharide (MDO) (33). *E. coli* DAG is also recycled to PA by DgkA (a functional homolog of DgkB) (48). This is unlike in eukaryotes, where Kennedy et al. demonstrated that PA is converted to both CDP-DAG (as in bacteria) and DAG by PA phosphohydrolase (PAP) enzymes, i.e., the reverse reaction catalyzed by Dgk enzymes (51, 52). In eukaryotes, DAG serves as a precursor for the synthesis of phosphatidylcholine (PC), phosphatidylethanolamine (PE), and phosphatidylserine (PS) (52). In both eukaryotes and bacteria, CDP-DAG serves as the precursor of phosphatidylinositol (PI), PG, and CL (48, 52). In eukaryotes, the *sn*-1,2 isoform of DAG also bears signaling properties not identified in bacteria (52). Rather, the accumulation of DAG in bacteria, if not recycled to PA, is toxic (33, 49, 53). DAG is a neutral lipid, and its accumulation in the membrane may cause disruption of the bilayer (54). In liposomes, DAG prevents the insertion of new polar lipids and membrane proteins (55). Thus, restricting the activity of LtaS at septal membranes leads to the local transient accumulation of this neutral lipid, which defines an asymmetry in the lipid composition of membranes.

To conclude, we reveal a mechanism by which LtaS activity is coupled to its processing and is governed by the engagement of the starter glycolipid unit Glc_2_-DAG. We hypothesize that upon engagement of Glc_2_-DAG with LtaS, SpsB is triggered to cleave the enzyme, releasing the catalytic domain eLtaS, which remains trapped in the septal compartment and continues to polymerize LTA. This creates a unique LTA-dependent environment, whereby DAG is preferentially enriched at septal membranes, thus favoring the recruitment of YSIRK precursors. Whether such recruitment requires additional protein factors remains to be determined.

## MATERIALS AND METHODS

### Bacterial strains and growth conditions

Tryptic soy broth (TSB) and agar (TSA) were used to grow *S. aureus* strains. Lysogeny broth (LB) and LB agar were used to grow *E. coli* strains. When necessary, spectinomycin, ampicillin, erythromycin, and chloramphenicol were used at 200, 100, 10, and 10 µg/mL, respectively. Isopropyl-β-D-1-thiogalactopyranoside and anhydrotetracycline were used at concentrations of 1 mM and 200 ng/mL, respectively.

### Plasmids and strains

The plasmids and strains used in this study are listed in Table S1, and the primers are listed in Table S2. RN4220 mutant strains lacking *ypfP* and *ltaA* were generated by transducing *bursa aurealis* transposon insertions from mutants ΦΝΞ171-39 and ANG359, respectively (30, 56), using phage ø85. The shuttle vector pSEW016 was used for complementation studies. pSEW016 is derived from pWWW412 (57) and carries the promoter and Shine-Dalgarno sequences of the *S. aureus hprK* gene. In pSEW016, the *Sac*I cloning site replaces the *Nde*I cloning site originally found in pWWW412. The *ypfP* and *ltaA* genes were amplified from genomic DNA prepared from strain RN4220 using the primer pairs YpfPWTF/YpfPWTR and LtaAWTF/LtaAWTR, respectively. A PCR product containing both genes *ypfP* and *ltaA* was generated by combining primers YpfPWTF and LtaAWTR since the two genes are adjacent on the chromosome. All PCR-amplified DNA fragments were cloned into the *Sac*I and *BamH*I restriction sites of pSEW016. Vector pKOR1 was used for allelic replacement as previously described (58). Briefly, 1-kb fragments upstream and downstream of the gene of interest were amplified by PCR. The following primers were used to (i) replace *cls1* with the spectinomycin cassette: primers Spec-F: 5′/Spec-R: 5′, attbCls1F1-F/Cls1F1-R, and Cls1F2-F/attbCls1F2-R; and (ii) generate the *cls2* knockout: primers PKCls2_F1/Cls2R-1 and Cls2F-2/PKCls2_R2. To generate the *cls1*/*cls2* double knockout, *cls1:spec* was transduced into the *cls2* knockout strain using phage ø85. All mutations were confirmed by sequencing.

### Microscopy

Microscopy experiments were performed mostly as described (21). Briefly, cultures were grown in the presence of appropriate antibiotics and inducers and collected at an *A*_600_ of 0.5 or otherwise at the indicated time points. Cells were pelleted by centrifugation for 3 min and washed once with phosphate-buffered saline (PBS), pH 7.4, and then subjected to sonication for 30 s to separate cells and avoid clustering. Cells were treated with trypsin 0.5 mg/mL (Sigma, USA) in 1 mL of PBS suspension and incubated with rotation for 1 h at 37°C. Following trypsin treatment, cells were washed twice with PBS and suspended in TSB with appropriate antibiotics/inducers and the soybean trypsin inhibitor at 2.5 mg/mL (Sigma, USA) prior to incubation at 37°C with rotation for 20 (*T*_20_) or 40 (*T*_40_) min. Cells were collected at *T*_20_ or *T*_40_ by centrifugation and suspended in PBS, and 250 µL was transferred to a new tube for immediate fixing in 2.5% paraformaldehyde and 0.006% glutaraldehyde in PBS. Fixing was performed at room temperature (RT) for 20 min, and then the cells were washed twice with PBS. Fixed cells were applied to eight-well poly-L-lysine-coated chamber coverslips (Ibidi, USA) and allowed to sit for 10 min. Excess cells were removed by suction and washed with PBS. Immobilized cells were blocked with 3% bovine serum albumin (BSA) in PBS for 1 h, followed by incubation with a SpA-specific humanized monoclonal antibody (1:20,000 dilution in 3% BSA/PBS) (59) overnight at 4°C. The next day, the cells were washed eight times with PBS, incubated with Alexa Fluor 594-conjugated anti-human IgG (1:500 in 3% BSA/PBS) (Invitrogen, USA) for 3 h at RT in the dark, washed 10 times with PBS, incubated with 1 µg/mL BODIPY FL-vancomycin (Invitrogen, USA) for 10 min at RT in the dark, and lastly washed five times with PBS. The slides were allowed to dry completely before adding ProLong Diamond Antifade Mountant (Invitrogen, USA). Fluorescence images were visualized and captured on a Leica Stellaris 8 confocal microscope with a 100x oil objective. Identical settings and laser intensities were applied to all samples.

### Culture and subcellular fractionation experiments and immunoblotting

Overnight bacterial cultures were diluted (1:100) into fresh TSB; when necessary, two subcultures were performed and grown at 37°C to *A*_600_ 0.5 or at otherwise indicated time points. To analyze proteins in whole cultures (WC), 1 mL was collected from each sample and immediately subjected to 20 µg/mL lysostaphin treatment for 30 min at 37°C, followed by 10% trichloroacetic acid (TCA) precipitation on ice for 1 h. Samples were centrifuged for 10 min at 20,000 × *g,* and precipitates were washed with ice-cold acetone and allowed to dry. For cell versus medium fractionation, 1.8 mL of sample cultures was transferred to an Eppendorf tube and spun at 20,000 × *g* for 5 min to separate cells in the pellet and the culture supernatant (labeled S in the figures). Proteins in S fractions were recovered by TCA precipitation (10% vol/vol). Cells in pellets were converted to protoplasts upon suspension in 1 mL TSM buffer [50 mM Tris (pH 7.5), 0.5 M sucrose, and 10 mM MgCl_2_] with treatment using 20 µg/mL lysostaphin for 10 min. Protoplast suspensions were either subjected to TCA precipitation to yield the total protein content of cells (labeled Cell in the figures) or spun at 20,000 × *g* for 10 min for subcellular fractionation. The new supernatants containing the cell wall fraction (labeled CW in the figures) were transferred to fresh tubes. Protoplasts were suspended in 1 mL Tris buffer [50 mM Tris-HCl (pH 7.5) and 10 mM MgCl_2_] and subjected to three freeze-thaw cycles in dry ice/ethanol and warm water baths. Centrifugation at 20,000 × *g* for 45 min at 4°C was used to separate the insoluble membrane fraction from the soluble cytosolic fraction, labeled M and C, respectively, in the figures. Proteins in all fractions were precipitated with 10% TCA as described and solubilized in 100 µL 1× SDS sample buffer [62.5 mM Tris-HCl (pH 6.8), 2% SDS, 10% glycerol, 5% 2-mercaptoethanol, and 0.01% bromophenol blue] and then boiled at 95°C for 10 min prior to SDS-PAGE analysis. Samples were separated on 12% (for LtaS) and 15% [for sortase A (SrtA)] SDS-PAGE and then transferred onto nitrocellulose membranes followed by western blotting. Membranes were blocked using 5% milk in TBST [50 mM Tris-HCl (pH 7.5), 150 mM NaCl, and 0.1% Tween 20] for 1 h in the presence of 50 µL human IgG (Sigma) to block non-specific binding of SpA. LtaS or SrtA rabbit polyclonal sera (laboratory reagents) were added to the blots (1:5,000 and 1:10,000 dilutions, respectively) and incubated overnight at 4°C. Next, the blots were washed three times for 5 min with TBST and then incubated with secondary anti-rabbit IgG linked to horseradish peroxidase (HRP) (Cell Signaling Technology, USA) for 1 h. The blots were washed three times with TBST, developed using SuperSignal West Pico Plus Chemiluminescence Substrate (ECL solution) (Thermo Scientific, USA), and visualized using a Photodyne imaging system.

### LTA extraction and immunoblotting

LTA was extracted mostly as described (23). Cultures were normalized to the same optical density, *A*_600_ of 3 for overnight cultures when comparing wild-type and mutant strains and *A*_600_ of 1 for time course experiments. One milliliter of cell suspensions was mixed with 0.2 g of glass beads, and bacteria were lysed by eight rounds of bead beating for 60 s each, with 5-min incubation periods on ice. Suspensions were centrifuged at 200 × *g* for 3 min to remove the glass beads, and 0.5 mL of the lysed cells was transferred to new tubes. Bacterial membranes and LTA were sedimented by centrifugation at 16,000 × *g* for 15 min. Pellets were resuspended in 1× SDS sample buffer and boiled for 10 min at 95°C. Samples were separated on 15% SDS-PAGE gels, followed by transfer to nitrocellulose membranes and western blotting. Blots were developed as mentioned, except that the mouse LTA-specific monoclonal antibody (1:200 dilution) (mAb 55 Novus Biologicals) was used as a primary antibody for LTA detection with secondary anti-mouse IgG linked to HRP (Cell Signaling Technology, USA).

### Lipid extraction and TLC quantification

Membrane lipids from *S. aureus* were extracted using a modified Bligh-Dyer method (37, 60). Cells were grown overnight in TSB in the presence of appropriate antibiotics when needed. One liter of cell culture was inoculated from the overnight cultures at a 1:100 dilution and allowed to grow at 37°C until OD_600_ of 0.8. Cells were collected and washed in 2% NaCl and then resuspended in 5 mL of 2% NaCl. Bacteria were then lysed using 20 µg/mL lysostaphin (AMBI, USA) at 37°C for 30 min. The lysed cell suspension was subjected to lipid extraction by the addition of a fivefold volume of chloroform-methanol (2:1, vol/vol) and mixed vigorously for 3 min and then left at RT for 10 min to settle. After 10 min, a threefold volume of chloroform-2% NaCl (1:1, vol/vol) was added, and the reaction was mixed and then spun down at 6,000 rpm for 15 min. The lower chloroform layer containing the lipids was recovered and concentrated under vacuum. Lipids were dissolved in chloroform-methanol (1:2, vol/vol) and applied to silica thin-layer chromatography plates (Silica gel 60 F_254_, Merck, Germany). Plates were developed with chloroform-methanol-acetic acid (65:25:10, vol/vol/vol) in a pre-equilibrated TLC developing chamber and then sprayed with 100 mg/mL CuSO_4_ solution containing 8% phosphoric acid and heated at 180°C to visualize the phospholipids. As controls, PG (14:0, sodium salt, Avanti, USA) and CL (14:0, sodium salt, Avanti, USA) were used as standards.

### Statistical analysis

For the analysis of cells using microscopy images, two different full fields of two biologically independent experiments were counted using ImageJ software (61). The images were used to enumerate SpA staining with restricted trafficking and normal display on the cell surface display versus aberrant SpA targeting or display. Typical patterns of SpA staining are depicted in Fig. 1C. Normal (restricted) versus aberrant Spa signal was calculated as a percentage of total cells counted. Data were analyzed using GraphPad Prism software, and two-way ANOVA (Tukey’s multiple comparison test) was used to compare the mean (SEM) for each mutant or complemented strain with the mean of wild-type cells. A similar analysis was performed for immunoblots, and the mean (SEM) was derived from three biologically independent repeats of subcellular fractionation experiments. LTA immunoblot signals were also analyzed from three independent experiments using one-way ANOVA (Dunnett’s multiple comparison test). TLC experiments were performed at least two times, and the mean (SEM) of scanned signals was analyzed with two-way ANOVA (Dunnett’s multiple comparison test).

## SUPPLEMENTAL MATERIAL

Supplemental figures and tables mbio.02852-23-s0001.pdf Fig. S1 and S2; Tables S1 and S2.

ASM does not own the copyrights to Supplemental Material that may be linked to, or accessed through, an article. The authors have granted ASM a non-exclusive, world-wide license to publish the Supplemental Material files. Please contact the corresponding author directly for reuse.
