# Supplementary material for: Processing of LtaS restricts LTA assembly and YSIRK preprotein trafficking into Staphylococcus aureus cross-walls
Source: mBio. 2024 Jan 4;15(2):e02852-23. doi: 10.1128/mbio.02852-23 (PMC10865820; doi:10.1128/mbio.02852-23)
Supplement: Supplemental figures and tables — Fig. S1 and S2; Tables S1 and S2. [file mbio.02852-23-s0001.pdf]

Supplemental material for

Processing of LtaS restricts LTA assembly and YSIRK preprotein trafficking into *S. aureus* cross-walls

Amany M. Ibrahim,[a,b] Muhammad S. Azam,[a] Olaf Schneewind,[a]† Dominique Missiakas[a]#

[a]Department of Microbiology, Howard Taylor Ricketts Laboratory, The University of Chicago, Lemont, Illinois, USA

[b]Department of Microbiology and Immunology, Faculty of Pharmacy, Sinai University, Arish, Egypt

Running Head: A trapping mechanism to restrict septal secretion

#Address correspondence to Dominique Missiakas, dmissiak@bsd.uchicago.edu

†Deceased.

Fig. S1

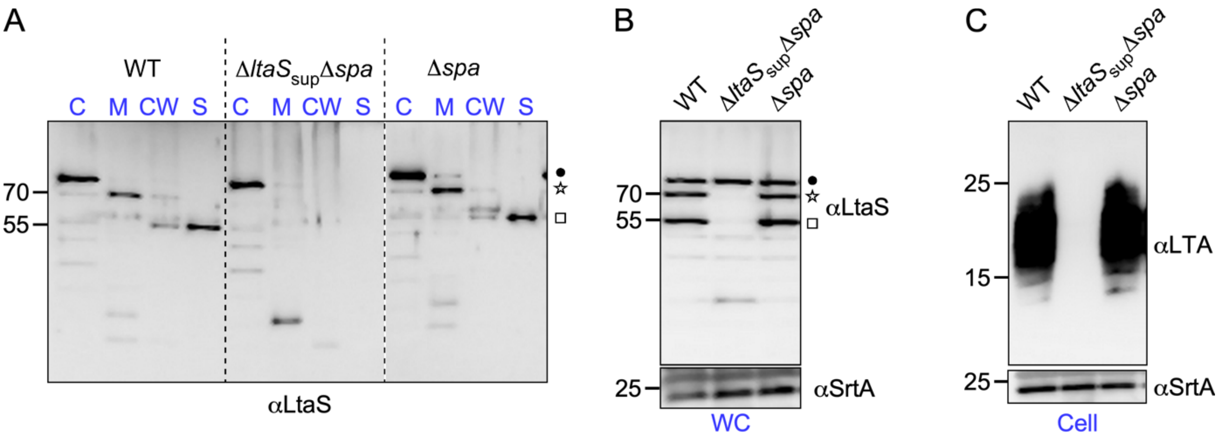

Fig. S1. Immunoblot analysis of LtaS. Bacterial cultures of WT(RN4220), Δ*ltaS*$_{sup}$Δ*spa* (RN4220 lacking *spa* and *ltaS* but carrying an extragenic suppressor mutation that restores growth), and an isogenic control Δ*spa* (RN4220 lacking *spa*) were normalized to the same optical density and were subsequently (**A**) fractionated into C, cytoplasm, M, membrane, CW, cell wall, and S, culture supernatant, (**B**) lysed directly, WC, whole culture, or (**C**) spun to isolate cells that were washed and subsequently lysed (Cell). Extracts were separated by SDS-PAGE and transferred to membranes for immunoblotting with anti-LtaS (αLtaS) or anti-SrtA (αSrtA, loading control) rabbit polyclonal sera or a monoclonal antibody against poly-GroP (αLTA). Star, square, and dot identify LtaS precursor (MW 70 kD) and mature protein (MW 55 kD), and an unknown protein cross-reactive, respectively. Only the cross-reactive species is observed in the□Δ*ltaS*$_{sup}$Δ*spa* extracts (**A**, **B**) which also fail to produce LTA (**C**). Numbers to the left of blots indicate the migration of molecular weight markers in kDa.

Fig. S2

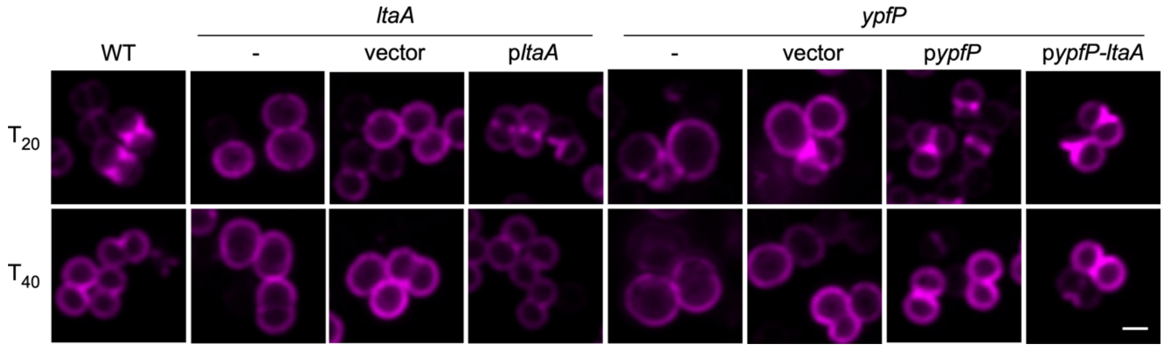

Fig. S2. Depleting the glycolipid anchor of LTA affects septal secretion of SpA. Images of *S. aureus* wild type (WT), *ltaA, ypfP* mutants, and their complemented strains shown in Fig. 2B are shown here with SpA staining only. Following trypsin removal of surface proteins, bacteria were allowed to recover for 20 and 40 min (T_{20}/T_{40}). Scale bars 2 μm.

Table S1. Bacterial strains or plasmids used in this study.

Name (Short name)[1]	Description	Source or reference
	Vectors and plasmids	
pSEW016	*E. coli/S. aureus* shuttle vector	Laboratory collection
pKOR1	Allelic replacement vector	(1)
pAMI-1 (p*ypfP*)	*ypfP* gene cloned into pSEW016	This study
pAMI-2 (p*ypfP-ltaA*)	*ypfP-ltaA* genes cloned into pSEW016	This study
pAMI-3 (p*ltaA*)	*ltaA* gene cloned into pSEW016	This study
pAMI-5 (pKOR-*cls1::spec)*	*cls1:spec* allele cloned into pKOR1	This study
pAMI-6 (pKOR1-Δ*cls2*)	*cls2* gene lesion cloned into pKOR1	This study
	**Strains**	
DH5α	*E. coli* cloning strain	Laboratory collection
RN4220 (WT, wild type)	*S. aureus* laboratory strain	(2)
SEJ1 (Δ*spa*)	RN4220 lacking *spa*	Laboratory collection
ANG1786 (Δ*spa*Δ*ltaS*$_{sup}$)	SEJ1 Δ*ltaS* with extragenic suppressor mutation restoring viability	(3)
AMI-7 (*ypfP)*	*Bursa aurealis* insertion in *ypfP* (*ypfP::erm* allele, [illegible]ΦNΞ171-39) transduced in RN4220	(4)
AMI-8 (*ltaA*)	*Bursa aurealis* insertion in *ltaA* (*ltaA::erm* allele) transduced in RN4220	(5)
AMI-9 (*ypfP*/p*ypfP*)	Strain AIM-7 complemented with pAIM-1	This study
AMI-10 (*ypfP*/p*ypfP-ltaA*)	Strain AIM-7 complemented with pAIM-2	This study
AMI-11 (*ltaA*/p*ltaA*)	Strain AIM-8 complemented with pAIM-3	This study
ANG499	RN4220 with IPTG-inducible *ltaS* expression	(6)
ANG513 (EL)	p*itet* integrated in strain ANG499	(6)
ANG514 (*ltaS*)	p*itet-ltaS* integrated in strain ANG499	(7)
ANG1121 (*ltaS*$_{T300A}$)	p*itet-ltaS*$_{T300A}$ integrated in strain ANG499	(7)
ANG1246 (*ltaS*$_{S218P}$)	p*itet-ltaS*$_{S218P}$ integrated in strain ANG499	(8)
AMI-15 (*cls1*)	RN4220 with in-frame deletion in *cls1*	This study
AMI-16 (*cls2)*	RN4220 with in-frame deletion in *cls2*	This study
AMI-17 (*cls1/csl2*)	RN4220 with in-frame deletion in *cls1* and *cls2*	This study

[1]Short names in parenthesis are used throughout this study to identify plasmids and strains described in the table.

Table S2. Oligonucleotide sequence of primers used in this study.

**Primer name**	**Purpose**	**Sequence**
YpfPWTF	*ypfP* complementation	5´GCGCGCGAGCTCATGGTTACTCAAAATAAAAAGATATTGATTATTACTGGC3´
YpfPWTR	*ypfP* complementation	5´GCGCGCGGATCCTTATTTAACGAAGAATCTTGCATATAAAGG3´
YpfPWTF	*ypfP* complementation	5´GCGCGCGAGCTCATGGTTACTCAAAATAAAAAGATATTGATTATTACTGGC3´
LtaAWTR	*ypfP* complementation	5´GCGCGCGGATCCTTACTTAGCTTTTTCTCTATTTACTATAAAGTAGC3´
LtaAWTF	*ltaA* complementation	5´GCGCGCGAGCTCATGGAAAGGTTCCTTTATATGCAAGATTCTTCG3´
LtaAWTR	*ltaA* complementation	5´GCGCGCGGATCCTTACTTAGCTTTTTCTCTATTTACTATAAAGTAGC3´
attbCls1F1-F	*cls1* allele replacement	5´GGGGACAAGTTTGTACAAAAAAGCAGGCTAACTCTTCCAATTCTGATTTAGAGTATAATGTGCC´3
Cls1F1-R	*cls1* allele replacement	5´GCGCTCGAGAGTCTTTTCTCCTATAAAGAAAGGC3´
Cls1F2-F	*cls1* allele replacement	5´GCGCCATGGTTTATTTGTAAGGAGTCTCGATTATAGAGGC3´
attbCls1F2-R	*cls1* allele replacement	5´GGGGACCACTTTGTACAAGAAAGCTGGGTCTTTTTGTATTTCAATATCATCC3´
Spec-F	Spec cassette	5´GCGCTCGAGATCGATTTTCGTTCGTGAATACATG3´
Spec-R	Spec cassette	5´GCGCCATGGGATATGCAAGGGTTTATTGTTTTCTAAAATCTG3´
PKCls2_F1	*cls2* allele replacement	5´GGGGACAAGTTTGTACAAAAAAGCAGGCTTCATCGAATAGTCCGACGATAGCTTTAACGG3´
Cls2R-1	*cls2* allele replacement	5´GCGCGAGCTCTTGAAACCTCCCATCGAAAATCTTAAACG3´
Cls2F-2	*cls2* allele replacement	5´GCGCGAGCTCAATAGAAATATGAGGAGTGTAACTTTAATGC3´
PKCls2_R2	*cls2* allele replacement	5´GGGGACCACTTTGTACAAGAAAGCTGGGTGCACATCAAAGTAACATGGCATCAACATCAAACCG3´

References:

1. T. Bae, O. Schneewind, Allelic replacement in Staphylococcus aureus with inducible counter-selection. *Plasmid* **55**, 58-63 (2006).
2. B. N. Kreiswirth *et al.*, The toxic shock syndrome exotoxin structural gene is not detectably transmitted by a prophage. *Nature* **305**, 709-712 (1983).
3. R. M. Corrigan, J. C. Abbott, H. Burhenne, V. Kaever, A. Grundling, c-di-AMP is a new second messenger in Staphylococcus aureus with a role in controlling cell size and envelope stress. *PLoS pathogens* **7**, e1002217 (2011).
4. T. Bae *et al.*, *Staphylococcus aureus* virulence genes identified by *bursa aurealis* mutagenesis and nematode killing. *Proc. Natl. Acad. Sci. USA* **101**, 12312-12317 (2004).
5. A. Gründling, O. Schneewind, Genes required for glycolipid synthesis and lipoteichoic acid anchoring in *Staphylococcus aureus*. *J. Bacteriol.* **189**, 2521-2530 (2007).
6. A. Gründling, O. Schneewind, Synthesis of glycerol phosphate lipoteichoic acid in *Staphylococcus aureus*. *Proc. Nat. Acad. Sci. USA* **104**, 8478-8483 (2007).
7. D. Lu *et al.*, Structure-based mechanism of lipoteichoic acid synthesis by Staphylococcus aureus LtaS. *Proc Natl Acad Sci U S A* **106**, 1584-1589 (2009).
8. M. E. Wörmann, N. T. Reichmann, C. L. Malone, A. R. Horswill, A. Gründling, Proteolytic cleavage inactivates the Staphylococcus aureus lipoteichoic acid synthase. *J Bacteriol* **193**, 5279-5291 (2011).
